# Awareness and Willingness for Body Donation in a Semi-urban Area of India: A Cross-Sectional Study

**DOI:** 10.7759/cureus.84565

**Published:** 2025-05-21

**Authors:** Stuti Shukla, Devendra S Shekhawat, Prabhjot K Chhabra, Abhilasha Maharshi, Mahindra Anand

**Affiliations:** 1 Medical School, Al-Falah School of Medical Sciences and Research Centre, Faridabad, IND; 2 Department of Anatomy, Institute of Medical Sciences, Banaras Hindu University, Varanasi, IND; 3 Anatomy, Mahatma Gandhi Medical College and Hospital, Jaipur, IND; 4 Department of Anatomy, Rama Medical College and Research Centre, Hapur, IND

**Keywords:** anatomy education, attitudes misconceptions, awareness campaign, cadavers, dead body donation

## Abstract

Background

The rising number of medical colleges in India has increased the demand for cadavers to support education on the subject of anatomy, which is a core component of medical training. However, societal, cultural, and religious barriers often limit body donation, resulting in an inadequate availability of cadavers.

Aim

This observational study aimed to assess awareness and willingness towards body donation among residents of Dhauj, a semi-urban area in the Faridabad district, and to identify key barriers to body donation.

Materials and methods

A pre-validated questionnaire was distributed to 524 participants and data were collected on their awareness, willingness, and misconceptions about body donation.

Results

Results indicate that 66.2% of participants had never heard of body donation, with only 26.3% expressing personal willingness to donate. Major barriers to donation included religious beliefs (39.8%), cultural norms (23.4%), and a lack of information (28.44%). Notably, family dynamics also impacted willingness, with 66.4% of participants uncertain about their families' support. A majority, 83.2% of respondents, placed trust in healthcare professionals as credible sources of information, emphasising the role of medical professionals in advocacy of dead body donation.

Conclusion

Results of the present study revealed a significant gap in awareness and willingness to participate in dead body donation within semi-urban communities. This study highlights the urgent need for targeted awareness campaigns, involvement of trusted health care professionals, community leaders and educational initiatives to address misconceptions about body donation and improve acceptance of body donation programs among residents of semi-urban areas of India.

## Introduction

Over the past decade, India has seen a substantial increase in the number of medical colleges, significantly expanding its medical education infrastructure as per the National Medical Commission document [1]. This expansion is particularly commendable given the country's large population, as it enhances access to medical education and meets the growing demand for healthcare professionals. Notably, India reached the WHO norm of a 1:1,000 doctor-population ratio in 2018 [2]. As the number of medical colleges has grown, so has the demand for cadavers to teach anatomy, the subject being a critical component of the medical curriculum.

Anatomy, the science of human biology, is a fundamental discipline essential for all medical and biomedical students [3]. It is widely regarded as a cornerstone of medical school curricula due to its crucial role in developing clinical skills [4]. A comprehensive understanding of anatomical structures is indispensable for performing safe and effective clinical practices. Learning anatomy through cadaver dissection promotes active and deep learning, allowing students to engage in tactile learning and simulate surgical skills, thereby enhancing their understanding of the relationship between patients' symptoms and underlying pathology [5].

Cadavers are invaluable resources in the anatomy curriculum, providing students with essential hands-on experience and a profound understanding of anatomical structures crucial for their foundational learning and future clinical practice [6]. Moreover, cadaveric studies contribute to advancements in surgical techniques and overall healthcare outcomes [7]. Availability of cadavers for dissection depends upon the dead body donation done by the general public under body donation programs. However, lack of availability of cadavers impedes effective anatomy education, creating a gap between theoretical knowledge and practical understanding [8]. This shortage is a significant challenge for Indian medical students and is exacerbated by a global cadaver shortage [9].

In 1949, India enacted the Anatomy Act, which was uniformly adopted across all states to facilitate the collection of deceased bodies for educational purposes in academic institutions [10]. Donation of a dead body is often surrounded by societal stigma and misconceptions, hindering its acceptance and practice [11]. We hypothesize that many individuals, particularly from lower socioeconomic classes and females, lack sufficient information about body donation and the posthumous donation process. This study aimed to assess awareness and willingness towards body donation, identify the barriers to body donation so as to propose specific strategies to facilitate dead body donation. There is critical need for comprehensive advertising and public awareness campaigns to increase knowledge and support for body donation.

## Materials and methods

An observational study was conducted among residents of the semi-urban area of Faridabad district after due permission from Al-Falah Institutional Ethics Committee (IEC-AFSMSRC/2025/119). Informed consent was obtained from each participant. Convenient sampling was done and residents of the semi-urban area aged 18 years and above who gave informed consent to participate in the study were included. Residents who were under 18 years of age or those who were above 18 but refused to give consent were excluded. Data collection was conducted in three weeks duration using a pre-validated questionnaire designed specifically for this research [12]. The questionnaire was designed to cover several aspects of body donation, including awareness of body donation for medical education purposes, perceptions about body donation, including cultural and religious views, personal willingness to donate one’s body after death, family attitudes towards body donation, and the sources of information participants had encountered about body donation.

Five hundred twenty-four participants individually completed the questionnaire with the help of volunteers to overcome language barriers. 

Data analysis

The demographic features of study participants were tabulated. All the data collected was analysed using Microsoft Excel 2019 (Microsoft, Redmond, WA, USA). The participants were categorised for their age, gender and educational status. Their awareness, willingness and perceptions about body donation were obtained to identify barriers in body donation. Data was analysed for frequency distribution to calculate the number of participants who agreed with a particular view mentioned in a closed-ended questionnaire. 

## Results

A total of 524 residents participated in the present study. Most of the participants (70.9%) were of the younger age group between 18 to 38 years. Out of 524, 52.1% (273) were males and 47.5% (248) were females. Only 43% were educated up to the primary level and only 29.9% had completed a secondary level of education. The remaining 46.95% did not receive any formal education (Table 1).

**Table 1:** Demographic profile of the subjects participated in the survey

CATEGORY	SUB-CATEGORY	PERCENTAGE
Age	18 to 38	70.99%
	39 to 58	24.43%
	59 & above	4.58%
Gender	Female	47.52%
	Male	52.10%
	Others	0.38%
Level of Education	Primary	14.12%
	Secondary	29.96%
	Graduate & above	8.97%
	No formal education	46.95%
Employment Status	Unemployed	51.53%
	Employed	48.47%
Socioeconomic Class	Upper	0.00%
	Upper middle	0.00%
	Lower middle	24.43%
	Upper lower	30.92%
	Lower	44.66%

Awareness about body donation

Only a few (33.8%) study participants from the suburban population have heard about body donation. Only 21.9% agreed that they had enough information about body donation. 19.8% agreed that they had some source of information to know bout body donation. Most of them heard about body donation from their community itself (7.3%).

Willingness to donate body

35.6% were open to the idea of donating their bodies in the future. 26.3% were personally willing to donate their body after death. 12.9% of participants believed that body donation would only be possible if their family agrees to donate their body after their death. 

Barriers and misconceptions

Religious beliefs were identified as the most common (39.8%) barrier in body donation, followed by cultural beliefs and lack of awareness. Twenty-five percent had a belief that donated bodies might be misused.

Attitude to spread awareness

80.8% of the participants agreed that medical professionals should spread awareness regarding body donation (Table 2).

**Table 2:** Awareness, attitude and perceptions of the study participants about body donation

S. NO.	Attribute		Yes (Percentage)	No (percentage)
1.	Awareness about body donation	Knew about dead body donation for medical studies	33.8	66.2
		One had enough information about body donation	21.9	78.1
		Had source of information to know about body donation	19.6	80.4
		a. Heard from community	7.3	
		b. Heard from hospitals	5.6	
		c. Heard from Educational institutions	3.9	
		d. Learned from social media	2.8	
2.	Willingness to donate dead body	If their families would donate their body after death	12.9	66.4 (20.6 were unsure)
		Open to the idea of donating their body in the future	35.6	64.4
		Personally willing to donate his/her body	26.3	73.7
3.	Barriers to donating dead body	Religious belief	39.8	8.0
		Cultural belief	23.4	
		Lack of awareness	28.4	
		Financial condition	0.4	
4.	Misconceptions about dead body donation	Donated bodies might be misused	25	75
		Respondents knew that body donation means giving one’s body for medical research and education after death	30.4	69.6
5.	Attitude to spread awareness about dead body donation	Participant would discuss body donation in their community	60.9	39.1
		Medical professionals should educate the public about body donation	80.8	19.2
		Trusted the information about body donation given by healthcare professionals.	83.2	16.8

## Discussion

The present study was undertaken to explore the level of awareness regarding body donation in the semi-urban region of Dhauj, Faridabad, Haryana. The study involved 524 participants with a diverse demographic distribution across age, gender and education level. The hypothesis driving this research was, the awareness about body donation among the local population would be limited, due to the influence of various religious, cultural, and educational factors.

Ballala et al. (2011) conducted a study at Kasturba Hospital, Manipal, South India, with 106 participants, revealing that 44% were aware of body donation. The present study shows that 33.8% of participants in the given locality had heard about body donation. The present study was conducted on the general population in a semi-urban district. That might be the reason for the low percentage of awareness found in the present study in comparison to the study done by Ballala et al., which primarily involved medical professionals and explored knowledge, attitudes, and practices regarding body donation within a medically informed population [13]. 32.1% of the general population was found to be aware of dead body donation in Maharashtra state, as revealed by a study conducted by Rokade and Gaikawad (2012) [14]. Four hundred participants from the general public were included in that study. This is comparable to the present study. Another study conducted by Parthasarathy (2018) found a significantly higher level of awareness about body donation (70%) [10]. However, their sample size was comparatively small (100 participants) and the demographic profile of participants is not mentioned in their publication.

7.6% of the participants mentioned community as source of information of awareness, 6% stated hospitals as source of awareness, 3.6% stated educational institutions as source of information and 2.6% learned from social media. 80.4% of participants who knew about body donation did not reveal any source of information of awareness. In contrast, a study conducted by Rokade and Gaikawad (2012) in Maharashtra found that the media served as the primary source of information about body donation for many participants [14].

The willingness of individuals and their families to participate in body donation reveals complex dynamics influenced by personal attitudes, family support, and awareness levels. The present study found that only 12.9% of participants believed their families would agree to donate their bodies after death, while 66.4% responded negatively, and 20.6% were uncertain. These results reflect similar findings from studies on organ donation willingness, such as the study by Chen et al. (2014) conducted in Taiwan [15], which highlighted that family support and attitudes were critical predictors of a family's willingness to consent to organ donation, particularly in situations involving ICU patients with irreversible conditions.

A relatively low number of participants (26.3%) expressed personal willingness to donate, while a more encouraging 35.6% were open to future consideration of body donation. These findings resonate with the above study’s [15] conclusions that both family support and a positive outlook on organ donation significantly impacted willingness to donate. Since families often play a decisive role in the consent process for body and organ donation, focusing on family-centric educational initiatives could foster greater acceptance. 

Religious beliefs were the primary barrier to body donation in present study, cited by 39.8% of respondents. This finding aligns with broader international research suggesting that religious and cultural values profoundly impact decisions on body donation. Another study [16] revealed that the scientific advancement of anatomy has largely sidelined religious and cultural considerations, despite these factors shaping individual willingness across diverse communities and faiths. Specifically, individuals within religious communities often hold views that emphasize the sanctity of the body, burial practices, and rituals that conflict with the idea of body donation. This hesitancy has been documented across multiple religions, including Islam, Hinduism, Buddhism, and Christianity, each with unique values and perspectives.

In the present study, cultural beliefs were also significant, with 23.4% of participants noting this as a barrier. This mirrors findings from communities with strong cultural ties, such as those within Confucian, Zulu, and Māori traditions, where specific customs and community values shape views on the body post-mortem [16]. Moreover, the 28.4% participants of the present study cited a lack of awareness as a barrier, indicating that limited exposure to information on body donation as a practice exacerbates existing religious and cultural concerns.

The study by Ballala et al. (2011) highlighted significant concerns among medical professionals regarding the misuse of donated bodies, with 85% expressing this belief. This apprehension may stem from a lack of knowledge about the ethical oversight of body donation, as many respondents were unaware of the authority governing the process or the criteria for acceptance. In comparison, the present study found that 25% of participants were worried about the potential misuse of bodies, suggesting a notable concern but indicating a lesser degree of anxiety compared to medical professionals [13].

Only 21.9% of respondents reported feeling adequately informed about body donation, while a substantial 78.1% expressed a lack of sufficient information. This is consistent with findings from a study conducted in Maharashtra [14], where only 32.1% of the general population was aware of body donation programs. 19.5% were willing to donate their bodies, indicating significant barriers rooted in misinformation and lack of awareness.

In the present study 83.2% of participants responded that they would trust information provided by healthcare professionals and 60.8% of respondents were willing to discuss body donation in their communities, emphasizing a potential for advocacy and education. The Taiwanese study also suggested the importance of healthcare professionals in educating families to overcome barriers to donation, an approach that may be effective in the present study as well. By engaging both potential donors and their families, healthcare providers could create a more informed and supportive environment, potentially increasing body donation willingness over time [15]. 80.8% of the study respondents believed that medical professionals should take an active role in public education about body donation. The reluctance to engage in body donation highlights the need for comprehensive awareness campaigns [14].

46.9% of participants in the present study had no formal education and 51.5% of respondents were unemployed. Jiang J et al studied demographic and motivational factors affecting whole body donation program, they found that body donors with an education level of college or above accounted for nearly half the deceased donors and donors working as teachers, government officials, medical staff and farmers formed a significantly larger proportion as compared to the general population [17]. Most participants in the present study belonged to lower socioeconomic classes, which may affect their perceptions of body donation.

Recommendations

Targeted awareness campaigns that explain body donation in simple, relatable terms to help the public understand its importance and impact can be developed. Training of healthcare professionals, especially doctors and nurses, to discuss body donation with patients as a standard part of care conversations should be done. This proactive approach can help normalize the topic and make patients more comfortable considering body donation. Involvement of respected community leaders and religious figures can help address cultural and religious questions surrounding body donation. Collaboration can be done with local media outlets to share personal stories and positive messages about body donation. Testimonials from donor families, healthcare professionals, and medical students who benefit from cadaveric study can be highlighted. Educational programs can be conducted involving discussions on body donation in schools and colleges. Age-appropriate materials can educate students on this vital contribution to medical science, fostering early understanding and positive attitudes toward donation.

Limitations

Although this study provides useful insights, there are some limitations to consider. First, the research was conducted in Dhauj, a semi-urban area in Faridabad, so the results may not apply to other regions with different cultures, socio-economic backgrounds, or education levels. The sample mainly consisted of people from lower educational and socio-economic backgrounds, which could affect how widely the findings can be applied. The reliance on self-reported data also means that participants might have given answers they think are more socially acceptable, rather than what they truly believe. The study didn’t measure how awareness campaigns actually affect people's views, which leaves room for further research in that area. Lastly, the impact of media campaigns, which could influence people's knowledge and opinions, wasn’t considered in this study.

## Conclusions

This study reveals significant gaps in awareness and willingness to participate in dead body donation within semi-urban communities, as shown by the findings from the present study. With over two-thirds of participants unaware of body donation and less than one-third expressing willingness to donate, cultural beliefs, religious views, and limited information emerge as primary barriers to acceptance. These insights highlight the need for focused strategies to improve understanding and acceptance of body donation. The data suggest that trusted healthcare providers can be effective advocates for the noble act of body donation.

## Appendices

Appendix

Annexure: Body Donation Questionnaire

The following questionnaire is designed to assess the awareness, beliefs, and willingness of individuals regarding body donation for medical research and education purposes. The responses will be used for academic research only and will be kept confidential. Your response will be crucial to design strategies to increase body donation.

SECTION A- Personnel Information

1.       Name - [Write your answer]
 

2.       Age - 18 to 28 - 29 to 38 - 39 to 48 - 49 to 58 - 59 & above
 

3.       Gender - Male - Female - Others
 

4.       Level of education - Primary - Secondary - Graduate & above - No formal education
 

5.       Employment status - Employed - Unemployed
 

6.      Monthly income - <10000 - 10000 to 20000 - 21000 to 30000 - 31000 to 40000 - >50000
 

Section B-  Please answer Q.7 to 17 in Yes/No

7.       Have you ever heard about giving your body for medical study after you pass away? - Yes - No
 

8.       I am aware that 'body donation' is defined as the act of giving one's body after death for medical research and education. - Yes - No
 

9.       In the event of your passing, would your family be willing to donate your body for scientific research or medical education purposes? - Yes - No - Not sure
 

10.   12. I believe that donated bodies are misused and mishandled. - Yes - No
 

11.   13. Do you believe that you have sufficient access to healthcare information regarding body donation? - Yes - No
 

12.   14. Do you trust the information provided by healthcare professionals regarding body donation? - Yes - No
 

13.   15. Will you discuss the topic of body donation with others in your community? - Yes - No
 

14.   16. Anatomy teaching and medical research can be done without cadavers. - Yes - No
 

15.   17. Medical professionals should explain and educate the general public about the importance of this noble act of 'body donation'. - Yes - No
 

16.   18. I can think about donating my body in the future. - Yes - No
 

17.   19. I am willing to donate my body. - Yes - No

SECTION C - Please provide honest response regarding-

18.   Where did you learn about body donation, if at all? - [Write your answer]
 

19.   Which one of these do you think is the barrier preventing you from body donation? - Lack of awareness - Financial crisis - Cultural beliefs - Religious beliefs
 
